# Morphological Subtypes of Tumor Spread Through Air Spaces in Non-Small Cell Lung Cancer: Prognostic Heterogeneity and Its Underlying Mechanism

**DOI:** 10.3389/fonc.2021.608353

**Published:** 2021-03-04

**Authors:** Huikang Xie, Hang Su, Erjia Zhu, Chang Gu, Shengnan Zhao, Yunlang She, Yijiu Ren, Dong Xie, Hui Zheng, Chunyan Wu, Chenyang Dai, Chang Chen

**Affiliations:** ^1^ Department of Pathology, Shanghai Pulmonary Hospital, Tongji University School of Medicine, Shanghai, China; ^2^ Department of Thoracic Surgery, Shanghai Pulmonary Hospital, Tongji University School of Medicine, Shanghai, China

**Keywords:** spread through air spaces, spread through a knife surface, non-small cell lung cancer, prognosis, artifact

## Abstract

**Background:**

Tumor spread through air spaces (STAS) has three morphologic subtypes: single cells, micropapillary clusters, and solid nests. However, whether their respective clinical significance is similar remains unclear.

**Methods:**

We retrospectively reviewed 803 patients with resected non-small cell lung cancer (NSCLC) from January to December 2009. Recurrence-free survival (RFS) and overall survival (OS) were compared among patients stratified by STAS subtypes. We also performed a prospective study of NSCLC resection specimens to evaluate the influence of a prosecting knife on the presence of STAS subtypes during specimen handling (83 cases).

**Results:**

STAS was found in 370 NSCLCs (46%), including 47 single cell STAS (13%), 187 micropapillary cluster STAS (50%), and 136 solid nest STAS (37%). STAS-negative patients had significantly better survival than patients with micropapillary cluster STAS (RFS: *P* < 0.001; OS: *P* < 0.001) and solid nest STAS (RFS: *P* < 0.001; OS: *P* < 0.001), but similar survival compared with those with single cell STAS (RFS: *P* = 0.995; OS: *P* = 0.71). Multivariate analysis revealed micropapillary cluster (RFS: *P* < 0.001; OS: *P* < 0.001) and solid nest STAS (RFS: *P* = 0.001; OS: *P* = 0.003) to be an independent prognostic indicator, but not for single cell STAS (RFS: *P* = 0.989; OS: *P* = 0.68). Similar results were obtained in subgroup analysis of patients with adenocarcinoma. The prospective study of NSCLC specimens suggested that 18 cases were considered as STAS false-positive, and most were singe cell pattern (13/18, 72%).

**Conclusions:**

Single cell STAS was the common morphologic type of artifacts produced by a prosecting knife. A precise protocol of surgical specimen handling is required to minimize artifacts as much as possible.

## Introduction

Tumor spread through air spaces (STAS) was added as a novel invasive pattern of lung adenocarcinoma (ADC) in the 2015 World Health Organization (WHO) classification (1). Subsequently, numerous studies consistently demonstrated STAS to be a prognostic risk factor for patients with ADC (2–12). This adverse impact extended to cases of squamous cell carcinoma (SQCC) and pleomorphic carcinoma, among others (13–16). Thus, STAS was recognized as a unique invasive type of non-small cell lung cancer (NSCLC) and attracted tremendous interests.

According to the 2015 WHO classification, STAS has three morphologic subtypes: single cells, micropapillary clusters, and solid nests. Our previous study showed that micropapillary cluster STAS was the most common type in ADC (6), and other studies found SQCC only featured solid nest STAS (13–15), which suggested the potential heterogeneity among STAS subtypes. Three STAS patterns were considered as one group in all published studies concerning clinicopathologic features and prognostic effect. Thus, it was unclear whether each subtype had distinct clinical behaviors.

In this study, we used a large retrospective cohort of patients with resected NSCLC to investigate the clinical characteristics of three STAS subtypes, with a focus on the survival outcomes. If differences among subtypes were observed, the potential mechanism was also explored.

## Materials and Methods

### Study Cohort

**Figure 1:**
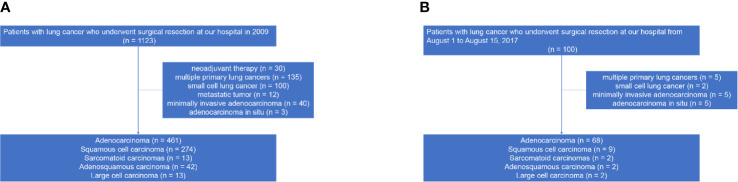
Study cohort flowchart. **(A)** Retrospective cohort; **(B)** Prospective cohort.

The institutional review board of Shanghai Pulmonary Hospital approved this study (No. K17-159). We reviewed 1,123 patients with lung cancer who underwent surgical resection at our hospital between January 1, 2009, and December 31, 2009. Patients with neoadjuvant therapy, multiple primary lung cancers, small cell lung cancer, metastatic tumor, minimally invasive adenocarcinoma, and adenocarcinoma *in situ* were excluded. After applying these criteria, a total of 803 patients with NSCLC were identified (**Figure 1A**). The tumors were classified according to the 2015 WHO classification and staged on the basis of the eighth edition of the TNM classification (1, 17). Patients’ clinical data were retrospectively extracted from electronic medical records. We also prospectively included 83 cases of NSCLC resection specimens from August 1, 2017 to August 15, 2017, according to the same inclusion and exclusion criteria to evaluate the influence of a prosecting knife on the presence of STAS subtypes during specimen handling (**Figure 1B**).

### Histopathologic Evaluation of STAS Subtypes

Tumor specimen slides were microscopically evaluated by two pathologists (H.X. and S.Z.) who were not aware of the clinical data. STAS was defined as tumor cells observed within air spaces in the surrounding lung parenchyma beyond the edge of the main tumor (1). The methods to distinguish STAS from artifacts and alveolar macrophages reported by Kadota et al. were adopted in this study (2). If diagnosis was still uncertain, immunohistochemistry for tumor cell marker (cytokeratin [AE1/AE3]) and macrophage marker (CD68) was performed.

**Figure 2:**
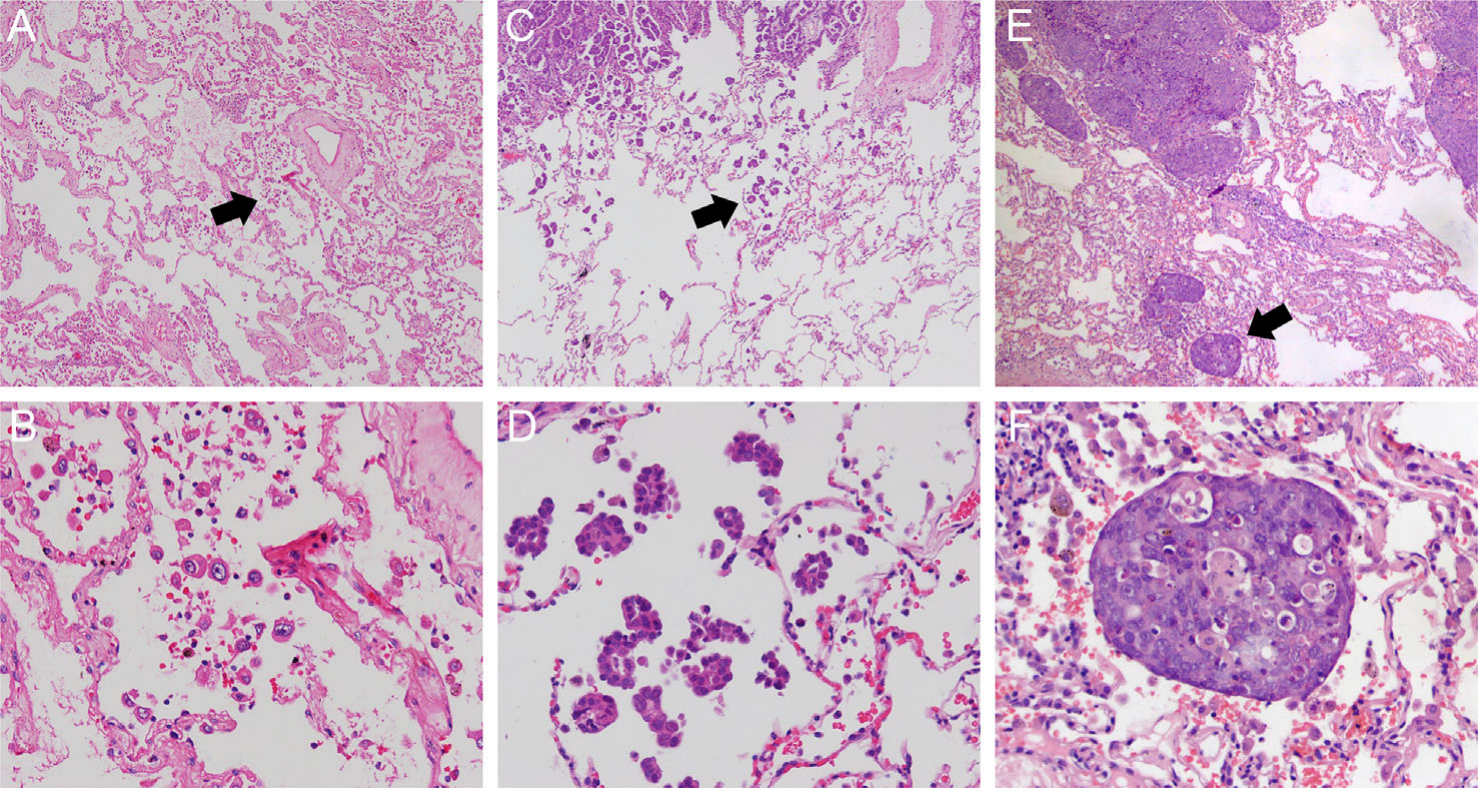
Morphologic features of *STAS* including single cell pattern (original magnification: ×40 in **(A)** and ×200 in **(B)** micropapillary cluster pattern (original magnification: ×40 in **(C)** and ×200 in **(D)** and solid nest pattern (original magnification: ×40 in **(E)** and ×200 in **(F)**. STAS, spread through air spaces**.

STAS has three morphologic patterns: (1) single cell pattern (**Figures 2A, B**), defined as discohesive single tumor cells within air spaces; (2) micropapillary cluster pattern (**Figures 2C, D**), defined as papillary structures without central fibrovascular cores filling as an alveolus; and (3) solid nest pattern (**Figures 2E, F**), defined as solid collections of tumor cells within an alveolus. Two pathologists (H.X. and S.Z.) categorized STAS into single cell, micropapillary cluster, or solid nest subtype independently. If any disagreement occurred, consensus was achieved after discussion.

### Survival Analyses for STAS Subtypes

The outcomes of interest were recurrence-free survival (RFS) and overall survival (OS), which were calculated using the Kaplan-Meier method and compared using the log-rank test among STAS subtype groups. Survival information was collected from outpatient clinic re-visit records (clinical, radiologic, and pathologic evaluation) and telephone follow-up through December 31, 2016. Multivariate survival analyses were conducted by using the Cox proportional hazards model to identify independent prognostic factors for RFS and OS. The variables were examined first using univariate analysis, and those with P value < 0.1 were incorporated into a multivariate model. We also assessed the prognostic significance of STAS subtypes in patients with ADC.

### Prospective Assessment of the Influence of a Prosecting Knife on STAS Subtypes

Two published studies suggested that STAS may partly be attributed to artifacts caused by a prosecting knife during specimen handling (18, 19). Our study also evaluated the influence of a prosecting knife on the presence of STAS subtypes. The same inclusion criteria used in the retrospective cohort were adopted to prospectively recruit patients with NSCLC who underwent surgery at our hospital between August 1, 2017, and August 15, 2017.

**Figure 3:**
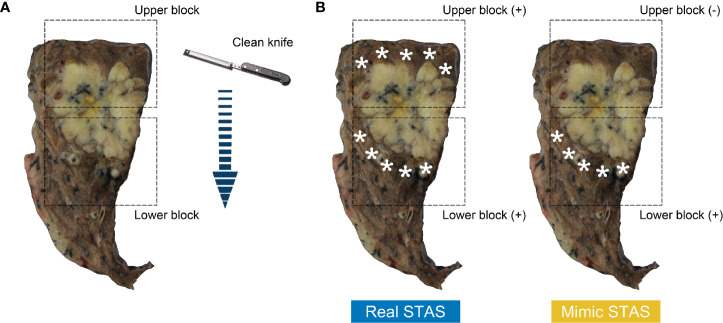
Surface of cross-section from resected lung specimen after the first cut **(A)**; arrow indicates cutting path. Tissue blocks in the rectangular box contains normal lung tissue above tumor (upper block) and below tumor (lower block). The diagrams of the definition of real STAS and mimic STAS **(B)**; pentagram indicates displaced tumor cells in normal lung tissue. STAS, spread through air spaces**.

The lung cancer specimens were prosected and sampled according to the following protocol (**Figure 3A**): (1) the lung cancer specimen was cut at its largest diameter using a clean, long prosecting knife, thus dividing the sample into two; (2) one tissue piece was randomly selected and divided into two sections along the vertical direction of the first cut by using a second clean knife; and (3) all specimens were cut in a single continuous direction to avoid excessive tumor tissue contamination caused by drawing the knife back and forth. Eventually, two tissue blocks were obtained. The upper block contained normal lung tissue and then tumor tissue, and the lower block contained tumor tissue and then normal lung tissue.

According to the cutting path, the normal lung tissue of the upper block was in contact with a clean blade, whereas that of the lower block was exposed to the blade after it made contact with tumor tissues. Hence, displaced tumor cells observed in the normal tissue of the lower block have the potential to theoretically be artifacts caused by contaminated blades. Morimoto and his colleagues found that free tumor clusters that had similar definitions of STAS were present in all directions of the main tumor (20). Therefore, cases could be considered as having real STAS when displaced tumor cells were identified in both upper and lower blocks, whereas cases were defined as having mimic STAS when displaced tumor cells were observed in the lower block but absent in the upper block (**Figure 3B**).

### Histopathologic Evaluation and Quantitative Comparison of STAS in Tissue Blocks

The surgically resected specimens were fixed with formalin, cut serially into 5-mm-thick slices, and macroscopically examined. Additional consecutive 4-µm-thick sections were cut from a selected tissue block and stained with hematoxylin and eosin. For each case, 5 to 10 tumor slides were reviewed. These slides were evaluated by two pathologists (H.X. and S.Z.) who were blinded to the information on sections and tissue blocks. The pattern and quantity of STAS were evaluated in each tissue block. The methodology was introduced in detail in a previous study (19). Briefly, all STAS in one visual field under a 10× objective were recorded as one occurrence, regardless of the absolute quantity of STAS in that field. The total number of STAS in the corresponding tissue block was estimated as the sum of all positive 10× objective fields in the H&E section. STAS with the largest number was considered the predominant subtype. If any disagreement occurred between the two reviewers, a third observer (C.W.) reviewed these slides.

### Statistical Analysis

All clinicopathologic data were presented as median (range), mean ± standard deviation, and number (percent). The Pearson χ^2^ test for categorical variables and Student *t* test or one-way ANOVA for numerical variables were applied to compare the groups. A two-sided P value of less than 0.05 was considered statistically significant. All analyses were performed using SPSS 22.0 (IBM Corporation, Armonk, NY) and GraphPad Prism 7.0 (GraphPad Software, San Diego, CA).

## Results

### Patient Characteristics

We identified 803 patients with NSCLC in the retrospective cohort. **Table 1** shows their detailed clinicopathological characteristics. Of these patients, 524 (65%) were men and 507 (63%) had no smoking history. The median age of this cohort was 60 years (range 29-91). ADC was the most common histological type (58%) (**Table 1**).

**Table 1:** Characteristics of patients with non-small cell lung cancer stratified by tumor spread through air spaces.

Variables	All patients	STAS (-)	STAS (+)	*P* value
	N = 803	N = 433	N = 370	
Age				
Median (range)	60 (29-91)	60 (29-91)	60 (33-82)	0.783
≤65	543 (68)	292 (67)	251 (68)	0.904
>65	260 (32)	141 (33)	119 (32)	
Gender				0.209
Male	524 (65)	291 (67)	233 (63)	
Female	279 (35)	142 (33)	137 (37)	
Smoking				0.049
Non-smoker	507 (63)	260 (60)	247 (67)	
Current or ex-smoker	296 (37)	173 (40)	123 (33)	
Carcinoembryonic antigen				<0.001
Normal	714 (89)	403 (93)	311 (84)	
High	89 (11)	30 (7)	59 (16)	
Tumor location				0.022
Upper & Middle	547 (68)	310 (72)	237 (64)	
Lower	256 (32)	123 (28)	133 (36)	
Surgical type				0.041
Limited resection	40 (5)	15 (4)	25 (7)	
Lobectomy	662 (82)	369 (85)	293 (79)	
Others	101 (13)	49 (11)	52 (14)	
Tumor histological type				<0.001
Adenocarcinoma	461 (58)	226 (52)	235 (64)	
Squamous cell carcinoma	274 (34)	178 (41)	96 (26)	
Others	68 (8)	29 (7)	39 (10)	
Tumor size				0.118
≤3 cm	465 (58)	265 (61)	200 (54)	
>3-5 cm	226 (28)	111 (26)	115 (31)	
≥5 cm	112 (14)	57 (13)	55 (15)	
Visceral pleural invasion				0.167
Absent	513 (64)	286 (66)	227 (61)	
Present	290 (36)	147 (34)	143 (39)	
Lymph node metastasis				<0.001
Negative	578 (72)	359 (83)	219 (59)	
N1 positive	47 (6)	17 (4)	30 (8)	
N2 positive	178 (22)	57 (13)	121 (33)	
Pathologic TNM stage				<0.001
Stage I	458 (57)	291 (67)	167 (45)	
Stage II	130 (16)	63 (15)	67 (18)	
Stage III/IV	215 (27)	79 (18)	136 (37)	
STAS Subtype				–
Single cell	47 (6)	–	47 (13)	
Micropapillary cluster	187 (23)	–	187 (50)	
Solid nest	136 (17)	–	136 (37)	
Postoperative chemotherapy				0.482
No	334 (42)	185 (43)	149 (40)	
Yes	469 (58)	248 (57)	221 (60)	

Values are presented as median (range) or n (%). STAS, spread through air spaces.

### Incidence and Features of STAS

Tumor STAS was identified in 370 of 803 patients (46%). STAS was more likely to be observed in patients with no smoking history (*P* = 0.049), elevated carcinoembryonic antigen (CEA) level (*P* < 0.001), ADC (*P* < 0.001), lymph node metastasis (*P* < 0.001) and high pathologic TNM stage (*P* < 0.001) (**Table 1**).

### Correlation of Clinicopathologic Characteristics with Different Types of STAS

When STAS was stratified by three morphologic patterns, 47 cases had single cell STAS (13%), 187 cases had micropapillary cluster STAS (50%), and 136 cases had solid nest STAS (37%) (**Table 2**). Large tumor size, lymph node metastasis, and high pathologic TNM stage were more frequently identified in tumors with micropapillary cluster STAS and solid nest STAS than those with single cell STAS (tumor size: *P* < 0.001; lymph node metastasis: *P* < 0.001; TNM stage: *P* = 0.003). In addition, female sex, no smoking history, and ADC were closely associated with the presence of single cell STAS and micropapillary cluster STAS, whereas male sex, a history of smoking, and SQCC were more common in tumors with solid nest STAS (gender: *P* < 0.001; smoking history: *P* < 0.001; histological type: *P* < 0.001) (**Table 2**).

**Table 2:** Characteristics of patients with non-small cell lung cancer stratified by subtypes of tumor spread through air spaces.

	Single cell STAS	Micropapillary cluster STAS	Solid nest STAS	*P* value
	N = 47	N = 187	N = 136	
Age				
Median (range)	59 (35-78)	61 (33-82)	61 (36-79)	0.335
≤65	37 (79)	124 (66)	90 (66)	0.232
>65	10 (21)	63 (34)	46 (32)	
Gender				<0.001
Male	18 (38)	102 (55)	113 (83)	
Female	29 (62)	85 (45)	23 (17)	
Smoking				<0.001
Non-smoker	37 (79)	143 (77)	67 (49)	
Current or ex-smoker	10 (21)	44 (23)	69 (51)	
Carcinoembryonic antigen				0.031
Normal	41 (87)	148 (79)	122 (90)	
High	6 (13)	39 (21)	14 (10)	
Tumor location				0.339
Upper & Middle	32 (68)	113 (60)	92 (68)	
Lower	15 (32)	74 (40)	44 (32)	
Surgical type				0.001
Limited resection	4 (9)	14 (7)	7 (5)	
Lobectomy	40 (85)	157 (84)	96 (71)	
Others	3 (6)	16 (9)	33 (24)	
Tumor histological type				<0.001
Adenocarcinoma	43 (92)	179 (96)	13 (10)	
Squamous cell carcinoma	2 (4)	1 (1)	93 (68)	
Others	2 (4)	7 (4)	30 (22)	
Tumor size				<0.001
≤3 cm	35 (74)	114 (61)	51 (38)	
>3-5 cm	8 (17)	59 (32)	48 (35)	
≥5 cm	4 (9)	14 (7)	37 (27)	
Visceral pleural invasion				0.01
Absent	30 (64)	101 (54)	96 (71)	
Present	17 (36)	86 (46)	40 (29)	
Lymph node metastasis				<0.001
Negative	38 (81)	93 (50)	88 (65)	
N1 positive	2 (4)	24 (13)	4 (3)	
N2 positive	7 (15)	70 (37)	44 (32)	
Pathologic TNM stage				0.003
Stage I	32 (68)	83 (44)	52 (38)	
Stage II	6 (13)	28 (15)	33 (24)	
Stage III/IV	9 (19)	76 (41)	51 (38)	
Postoperative chemotherapy				0.732
No	21 (45)	76 (41)	52 (38)	
Yes	26 (55)	111 (59)	84 (62)	

Values are presented as median (range) or n (%). STAS, spread through air spaces.

### Survival Analyses

**Figure 4:**
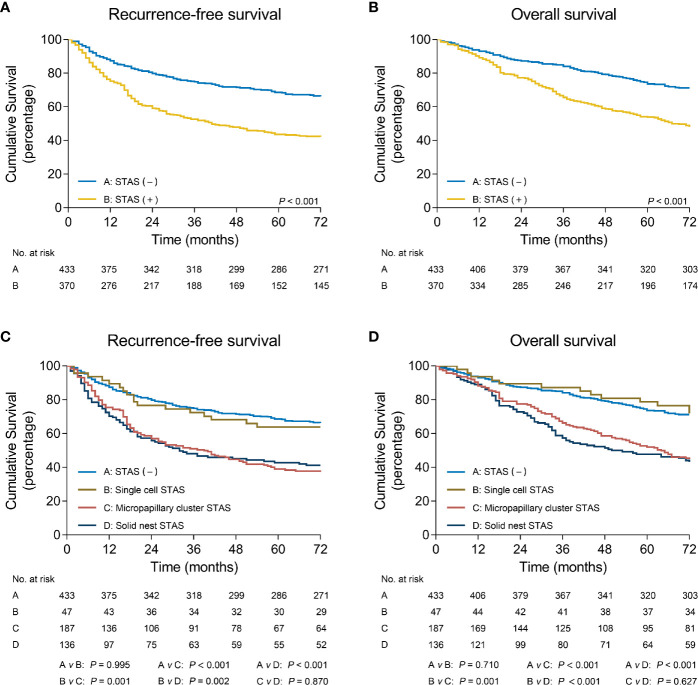
Recurrence-free survival **(A)** and overall survival **(B)** in patients with non-small cell lung cancer stratified by STAS. Recurrence-free survival **(C)** and overall survival **(D)** in patients with non-small cell lung cancer stratified by STAS subtypes. STAS, spread through air spaces**.

**Figures 4A, B** shows that patients without STAS had better RFS (*P* < 0.001) and OS (*P* < 0.001) than those with STAS. When stratifying STAS-positive patients by morphologic subtypes, patients without STAS had significantly better survival than did patients with micropapillary cluster STAS (RFS: *P* < 0.001; OS: *P* < 0.001) and solid nest STAS (RFS: *P* < 0.001; OS: *P* < 0.001), but comparable survival to that of patients with single cell STAS (RFS: *P* = 0.995; OS: *P* = 0.71) (**Figures 4C, D**).

In addition, multivariate analyses confirmed that the presence of micropapillary cluster STAS (RFS: hazard ratio [HR] = 1.75, 95% confidence interval [CI]: 1.30-2.37, *P* < 0.001; OS: HR = 1.99, 95% CI: 1.44-2.76, *P* < 0.001) and solid nest STAS (RFS: HR = 1.60, 95% CI: 1.21-2.14, *P* = 0.001; OS: HR = 1.55, 95% CI: 1.16-2.07, *P* = 0.003) was indicated as an independent prognostic factor, but the presence of single cell STAS was not (RFS: HR = 1.00, 95% CI: 0.59-1.70, *P* = 0.989; OS: HR = 1.13, 95% CI: 0.63-2.03, *P* = 0.68) (**Table 3**).

**Table 3:** Cox proportional hazards regression model for recurrence-free survival and overall survival in patients with non-small cell lung cancer.

Variables	Recurrence-free survival			Overall survival		
	Univariate Analysis	Multivariate Analysis		Univariate Analysis	Multivariate Analysis	
	*P* value	HR (95% CI)	*P* value	*P* value	HR (95% CI)	*P* value
Age						
>65 vs. ≤65	0.036	1.19 (0.96-1.48)	0.113	<0.001	1.48 (1.19-1.84)	<0.001
Gender						
Female vs. Male	0.177			0.004	0.71 (0.55-0.92)	0.009
Smoking						
Current or ex-smoker vs. Non-smoker	0.372			0.159		
Carcinoembryonic antigen						
High vs. Normal	<0.001	1.75 (1.33-2.30)	<0.001	<0.001	1.83 (1.38-2.41)	<0.001
Tumor location						
Lower lobe vs. Upper & middle lobe	0.262			0.45		
Surgical type						
Lobectomy & others vs. Limited resection	0.331			0.066	0.45 (0.29-0.71)	0.001
Tumor histological type						
SQCC & others vs. Adenocarcinoma	0.013	1.30 (0.97-1.75)	0.085	<0.001	1.63 (1.18-2.24)	0.003
Tumor size	<0.001		<0.001	<0.001		0.007
3-5 cm vs. ≤3cm	0.002	1.14 (0.90-1.45)	0.281	<0.001	1.26 (0.98-1.62)	0.073
≥5 cm vs. ≤3cm	<0.001	1.86 (1.39-2.50)	<0.001	<0.001	1.62 (1.19-2.21)	0.002
Visceral pleural invasion						
Present vs. Absent	0.013	1.17 (0.94-1.47)	0.168	0.017	1.36 (1.07-1.72)	0.012
Lymph node metastasis						
Positive vs. Negative	<0.001	2.48 (1.99-3.11)	<0.001	<0.001	2.48 (1.96-3.14)	<0.001
STAS Subtype	<0.001			<0.001		<0.001
Single cell STAS vs. Negative	0.998	1.00 (0.59-1.70)	0.989	0.717	1.13 (0.63-2.03)	0.68
Micropapillary cluster STAS vs. Negative	<0.001	1.75 (1.30-2.37)	<0.001	<0.001	1.99 (1.44-2.76)	<0.001
Solid nest STAS vs. Negative	<0.001	1.60 (1.21-2.14)	0.001	<0.001	1.55 (1.16-2.07)	0.003
Postoperative chemotherapy						
Yes vs. No	0.45			0.485		

HR, hazard ratio; CI, confidence interval; STAS, spread through air spaces.

### Subgroup Analysis of Patients with ADC

We also assessed the clinical significance of STAS subtypes in patients with ADC. Similar results were acquired in this subgroup when compared with those in entire cohort.

Tumor STAS was identified in 235 patients with ADC (51%), including 43 cases with single cell STAS (18%), 179 cases with micropapillary cluster STAS (76%), and 13 cases with solid nest STAS (6%) (**Supplementary Table 1**). The proportions of lymph node metastasis and high pathologic TNM stage were greater in tumors with micropapillary cluster STAS and solid nest STAS than in those with single cell STAS (lymph node metastasis: *P* = 0.003; TNM stage: *P* = 0.025). (**Supplementary Table 2**) Single cell STAS was observed in lepidic (11/103, 11%), acinar (17/224, 8%), papillary (12/85, 14%) and solid (3/37, 8%) predominant ADC, except for micropapillary predominant ADC. Micropapillary cluster STAS was observed in lepidic (14/103, 14%), acinar (104/224, 46%), papillary (34/85, 40%) and solid (16/37, 43%) predominant ADC. Interestingly, micropapillary STAS had a significant association with micropapillary predominant ADC (11/12, 92%). Whereas solid nest STAS was more common in patients with solid predominant ADC (Lepidic: 2/103, 2%; Acinar: 4/224, 2%; Papillary: 1/85 1%; Micropapillary: 0/12, 0%; Solid: 6/37, 16%).

**Supplementary Figures 1A, B** shows that STAS significantly stratified the RFS (*P* < 0.001) and OS (*P* < 0.001) in patients with ADC. Further analyses indicated that, when compared to patients with ADC without STAS, similar survival outcomes were found in those with ADC with single cell STAS (RFS: *P* = 0.639; OS: *P* = 0.708), but worse survival outcomes in those with ADC with micropapillary cluster STAS (RFS: *P* < 0.001; OS: *P* < 0.001) or with solid nest STAS (RFS: *P* < 0.001; OS: *P* = 0.002) (**Supplementary Figures 1C, D**). Multivariate analyses revealed micropapillary cluster STAS (RFS: HR = 1.67, 95% CI: 1.18-2.37, *P* = 0.004; OS: HR = 1.73, 95% CI: 1.19-2.51, *P* = 0.004) and solid nest STAS (RFS: HR = 2.13, 95% CI: 1.02-4.45, *P* = 0.043; OS: HR = 2.09, 95% CI: 0.95-4.63, *P* = 0.068) to be a risk factor for survival, but single cell STAS was not (RFS: HR = 0.82, 95% CI: 0.45-1.49, *P* = 0.517; OS: HR = 0.94, 95% CI: 0.48-1.83, *P* = 0.843) (**Supplementary Table 3**).

### Influence of a Prosecting Knife on STAS Subtypes

Because single cell STAS was not a significant prognostic factor, we next verified the hypothesis that single cell STAS was the artifact caused by a prosecting knife during specimen handling. A total of 83 patients with NSCLC who underwent surgery at our department met the inclusion criteria. **Supplementary Table 4** shows baseline characteristics of patients and pathologic results of tumors. All lung cancer specimens were prosected and sampled according to the standard protocol.

### Incidence and Features of STAS in Tissue Blocks

**Figure 5:**
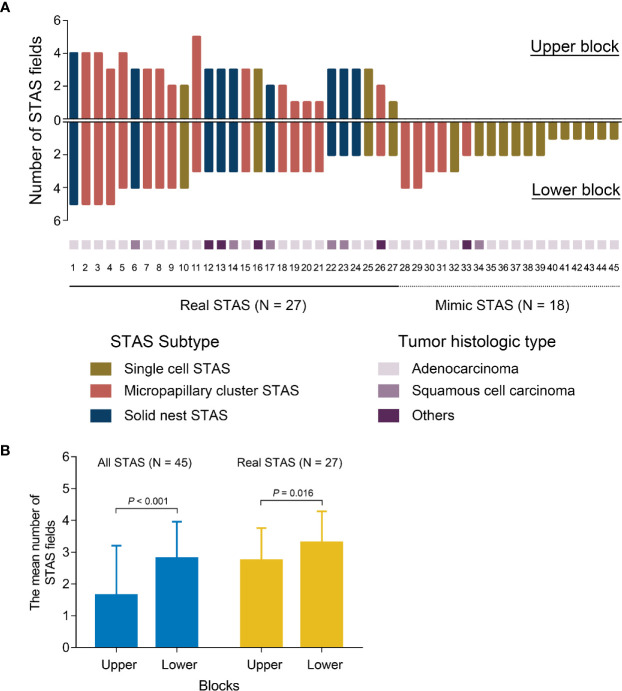
The distribution and quantity of STAS in each tissue block **(A)**. The quantitative comparison of all STAS and real STAS between upper blocks and lower blocks **(B)**. STAS, spread through air spaces**.

After histologic evaluation, 45 of 83 patients (54%) had displaced tumor cells in at least one tissue block (**Figure 5A**). The mean fields of displaced tumor cells were significantly greater in the lower part of the cuts than in the upper part (*P* < 0.001) (**Figure 5B**). Of these 45 patients, 27 (60%) were identified as having displaced tumor cells in both two blocks and diagnosed as having real STAS. The remaining 18 (40%) had displaced tumor cells in lower block but not in upper block; they were considered to have mimic STAS (**Figure 5A**). In patients with real STAS, upper blocks still had fewer fields of STAS compared to lower blocks (*P* = 0.016) (**Figure 5B**). In patients with mimic STAS, a great number of displaced tumor cells presented as single cell pattern (13/18, 72%) and in ADCs (16/18, 89%).

### Distribution of STAS Stratified by Morphologic Subtype

When subclassifying cases according to the morphologic features of STAS, 17 cases had single cell pattern, 19 cases had micropapillary pattern, and 9 cases had solid nest pattern (**Supplementary Figure 2**).

**Supplementary Figure 2A** shows the distribution of single cell STAS in 17 cases; the lower blocks had significantly more displaced tumor cells than the corresponding upper blocks (*P* < 0.001) (**Supplementary Figure 2B**). Of these 17 cases, 4 cases (24%) with real STAS and 13 cases (76%) with mimic STAS. No statistical difference in the number of positive fields was observed between upper blocks and lower blocks in patients with real single cell STAS (*P* = 0.495) (**Supplementary Figure 2B**).

Among 19 patients with micropapillary cluster STAS, 14 patients were considered as having real STAS (79%), and the remaining 5 patients had mimic STAS (21%) (**Supplementary Figure 2C**). The number of micropapillary cluster STAS fields in lower blocks was significantly higher than that in upper blocks in all cases (*P* < 0.001) and in cases with real STAS (*P* = 0.009) (**Supplementary Figure 2D**).

A solid nest pattern was observed in 9 cases (**Supplementary Figure 2E**). All patients (100%) had STAS in upper blocks and thus were considered as having real STAS. The number of positive fields of solid nest STAS was similar between upper and lower blocks (*P* = 0.998) (**Supplementary Figure 2F**).

## Discussion

To the best of our knowledge, this is the first study to examine the clinical significance of three STAS patterns. Our results suggested that unlike micropapillary cluster STAS and solid nest STAS, single-cell STAS was not significantly associated with pathologic features of aggressive tumor behavior (larger tumor size, lymph node metastasis, and high TNM stage). More importantly, the presence of single-cell STAS failed to stratify the prognosis in the study cohort, whereas micropapillary cluster STAS and solid nest STAS were confirmed as independent prognostic factors for both RFS and OS. Similar results were found in the subgroup of patients with ADC. Evidence of heterogeneity among STAS subtypes raises the question of whether single-cell STAS occurs as a mechanical artifact caused by specimen processing. Our prospective study of resected specimens verified that a prosecting knife blade disseminated tumor cells into normal lung tissues, thus leading to mimic STAS, which mostly presented as a single-cell pattern (72%).

Kadota et al (2). first defined STAS and reported its clinical significance in lung ADCs in 2015. They also reported three morphological patterns of STAS: (1) micropapillary structures consisting of papillary structures without central fibrovascular cores that occasionally form ring-like structures within air spaces; (2) solid nests or tumor islands consisting of solid collections of tumor cells filling air spaces; and (3) single cells consisting of scattered discohesive single cells. In addition, our previous study reported that STAS was always identified simultaneously with high-grade histologic patterns. Specifically, STAS occurred less frequently in lepidic-predominant ADC and more frequently in micropapillary and solid-predominant subtypes. However, few studies have investigated whether the three patterns of STAS have different features and correlations with pathologic subtypes of lung ADC. We found that micropapillary cluster STAS was more prevalent than single-cell STAS in every subtype of ADC. Furthermore, our results revealed that single-cell STAS failed to stratify the prognosis in the study cohort. Only micropapillary cluster STAS and solid nest STAS were independent prognostic factors for both RFS and OS. This is the first report about the prognostic impact of the three subtypes of STAS. This result indicated that single-cell STAS may occur as a mechanical artifact caused by specimen processing.

Since the introduction of STAS in 2015, many retrospective studies have unanimously shown its clinical and prognostic value in all major histologic types of NSCLC (2–16), proving that STAS is a biological phenomenon. Even with such sufficient published evidence, STAS is still controversial (18, 19, 21). Thunnissen and colleagues found that tumor fragments and individual cells could be spread into normal lung tissues through a knife surface (STAKS) and suggested that STAS might be an artifact (18). In the present study, we identified the possibility that most single-cell STAS could be artifacts because they lacked clinical and prognostic value. We then validated this speculation. These results have several important implications. First, single-cell STAS was the most common diagnostic pitfall and should be diagnosed very cautiously in retrospective studies. Generally, detailed records of specimen handling were unavailable in retrospective studies; thus, the potential effect of STAKS could not be eliminated. Second, a precise protocol of surgical specimen handling will be required to minimize artifacts as much as possible.

The key question that led to the speculation of STAS being an artifact rather than an invasive pattern was the survival of the tumor cells after detaching from the main tumor and floating freely in the air spaces without a vascular supply. Onozato and colleagues used an algorithm for 3-dimensional reconstruction of paraffin-embedded tissues and found that tumor islands (similar to the solid nest pattern) were connected to each other and to the main tumor at different levels, supporting the possibility that tumor islands gain access to energy supply from the main tumor (22). In a recent study, a high-quality 3-dimensional reconstruction and multiplex immunofluorescence study reported by Yagi and her colleagues revealed that micropapillary structures in normal air spaces that appeared to be free floating on 2-dimensional evaluation were actually attached to alveolar walls and capillaries through vessel cooption on 3-dimensional evaluation, thus gaining access to an energy supply (23). The study strongly support the hypothesis that solid nest STAS and micropapillary cluster STAS represent intraparenchymal invasion rather than artifacts, which is consistent with our findings. However, how single tumor cells can survive within air spaces remains unclear. If tumor cells can obtain access to an energy supply by adhering to the alveolar wall, individually scattered tumor cells suspended in the alveolar spaces seem to lack an energy supply and thus would hypothetically have difficulty surviving, which supports our findings that most displaced single tumor cells were artifacts rather than invasive growth.

Our results indicated that knife blades caused a small number of false-positive STAS cases with a micropapillary cluster pattern (28%). Yagi and colleagues found that micropapillary structures within airspaces in the main tumor area were connected to alveolar walls (23). Our findings suggested that the adhesive force was weak and could be easily broken by a knife. A similar phenomenon was reported by Isaka and colleagues (24). They found that micropapillary clusters could be aspirated out within airway secretions from the bronchus in which the tumor was located. More importantly, our results also revealed that a knife blade increased the number of micropapillary clusters in tumors with real STAS. Recently, Uruga and colleagues reported a semiquantitative assessment of STAS based on a retrospective analysis of 208 cases (5). Patients with early-stage ADC could be classified into high-STAS (≥ 5 single cells or clusters), low-STAS (1-4 single cells or clusters) and no-STAS groups. The survival analyses indicated that the high-STAS group was associated with worse RFS than the low-STAS and no-STAS groups. Nevertheless, considering that STAKS was neglected in this retrospective study, the possibility that STAS was overestimated cannot be entirely ruled out. For this reason, this semiquantitative method should be better verified in prospective studies.

Our results showed that the knife blade only slightly changed the frequency and quantity of displaced tumor cells with a solid nest pattern; thus, STAKS probably had little influence on findings related to solid nest STAS. Three retrospective studies investigated the prognostic implications of STAS in 445, 216, and 220 patients with SQCC, and all STAS-positive cases showed a solid nest pattern and were significantly associated with worse survival outcomes (13–15). Consequently, the prognostic value of STAS in SQCCs is still trustworthy even when STAKS is not taken into consideration.

Some limitations of this study should be addressed. First, this was a single-center study with some potential biases, and the results should be externally validated. Second, we proved the mechanical influence of a knife blade on STAS, but one could reasonably speculate that there might be additional mechanical forces on a tumor during specimen handling; thus, further studies are needed to explore their roles in the spread of tumor cells. Finally, the retrospective cohort and prospective cohort were two individual cohorts from 2009 and 2017, respectively. For the retrospective cohort, STAKS could not be evaluated because tumor specimens were processed following routine clinical protocols in 2009. For the prospective cohort, the results of survival analysis are not reliable for patients because of the short follow-up time. Thus, the prognostic impact of STAKS cannot be directly validated. Despite this limitation, the results of our study could provide some important information. Our data showed that single-cell STAS was not a prognostic factor and that a large proportion of single-cell STAS could be artifacts. This result indicated that the nonsignificant prognostic result of single-cell STAS was caused by single-cell STAKS. A precise protocol to eliminate single-cell STAKS should be designed in the future. Micropapillary cluster STAS and STAKS were highly associated with the presence of micropapillary components. This result indicated that micropapillary STAS may be cell clusters from micropapillary components in lung adenocarcinoma. Although some micropapillary STAS could be caused by a prosecting knife, the result also indicated the presence of a micropapillary component in lung adenocarcinoma. The presence of micropapillary clusters in airspaces merely reflects the aggressive biology of the tumor and dictates patient outcomes, irrespective of whether the clusters are real or artifacts (25).

## Conclusions

The presence of micropapillary cluster STAS and solid nest STAS were independent prognostic factors for shortened survival. However, single-cell STAS did not have prognostic significance, and most might be contaminants produced by a prosecting knife. Thus, single-cell STAS should be diagnosed very cautiously in retrospective studies because detailed records of specimen handling are generally unavailable to eliminate the potential effect of STAKS. In addition, a precise protocol of surgical specimen handling is required to minimize artifacts as much as possible.

## Data Availability Statement

The original contributions presented in the study are included in the article/**Supplementary Material**. Further inquiries can be directed to the corresponding authors.

## Ethics Statement

This study was carried out in accordance with the principles of the Helsinki Declaration of the World Medical Association. The study protocol was approved by the Institutional Review Board of Shanghai Pulmonary Hospital (No. FK-17-159).

## Author Contributions

(I) Conception and design: HX, CD, CW, and CC. (II) Administrative support: CW and CC. (III) Provision of study materials or patients: HS, EZ, and CG. (IV) Collection and assembly of data: SZ, YS, YR, DX, and HZ. (V) Data analysis and interpretation: HX, CD. (VI) Manuscript writing: All authors. All authors contributed to the article and approved the submitted version.

## Funding

Supported by the grants from National Natural Science Foundation of China (NSFC 9195910169 and NSFC 81770091 and NSFC 81902335 and NSFC 81802256) and the “Chen Guang” project supported by Shanghai Municipal Education Commission and Shanghai Education Development Foundation (18CG19) and the “Outstanding young talent” project supported by Shanghai Pulmonary Hospital (FKYQ1907), Shanghai Rising Star Program (20QA1408300) and Clinical Research Plan of SHDC (SHDC2020CR4028 and SHDC2020CR1021B) and National Key Research and Development Project (2019YFE0101200), Shanghai Science and Technology Committee (20YF1441100 and 20XD1403000 and 18DZ2293400), Shanghai Municipal Health Commission (2019SY072 and 2018ZHYL0102), Shanghai Pulmonary Hospital Innovation group project (Shanghai pulmonary hospital Innovation group project–“Chang Chen”) the Clinical Research Project of Shanghai Pulmonary Hospital (FK18001 and FK1904 and FKGG1805 and FK1936 and FK1943 and FKLY20007 and FKCX1906), Clinical Research Foundation of Shanghai Pulmonary Hospital (FK1944).

## Conflict of Interest

The authors declare that the research was conducted in the absence of any commercial or financial relationships that could be construed as a potential conflict of interest.

## Supplementary Material

Supplementary Figure 1 Recurrence-free survival (A) and overall survival (B) in patients with adenocarcinoma stratified by STAS. Recurrence-free survival (C) and overall survival (D) in patients with adenocarcinoma stratified by STAS subtypes. STAS, *spread through air spaces*.

Supplementary Figure 2 The distribution and quantity of STAS subtypes in each tissue block (A, single cell; C, micropapillary cluster; E, solid nest). The quantitative comparison of all STAS subtypes and real STAS subtypes between upper blocks and lower blocks (B, single cell; D, micropapillary cluster; F, solid nest). STAS, *spread through air spaces*.
