# Supplementary figures and images for: Morphological Subtypes of Tumor Spread Through Air Spaces in Non-Small Cell Lung Cancer: Prognostic Heterogeneity and Its Underlying Mechanism
Source: Front Oncol. 2021 Mar 4;11:608353. doi: 10.3389/fonc.2021.608353 (PMC7970243; doi:10.3389/fonc.2021.608353)

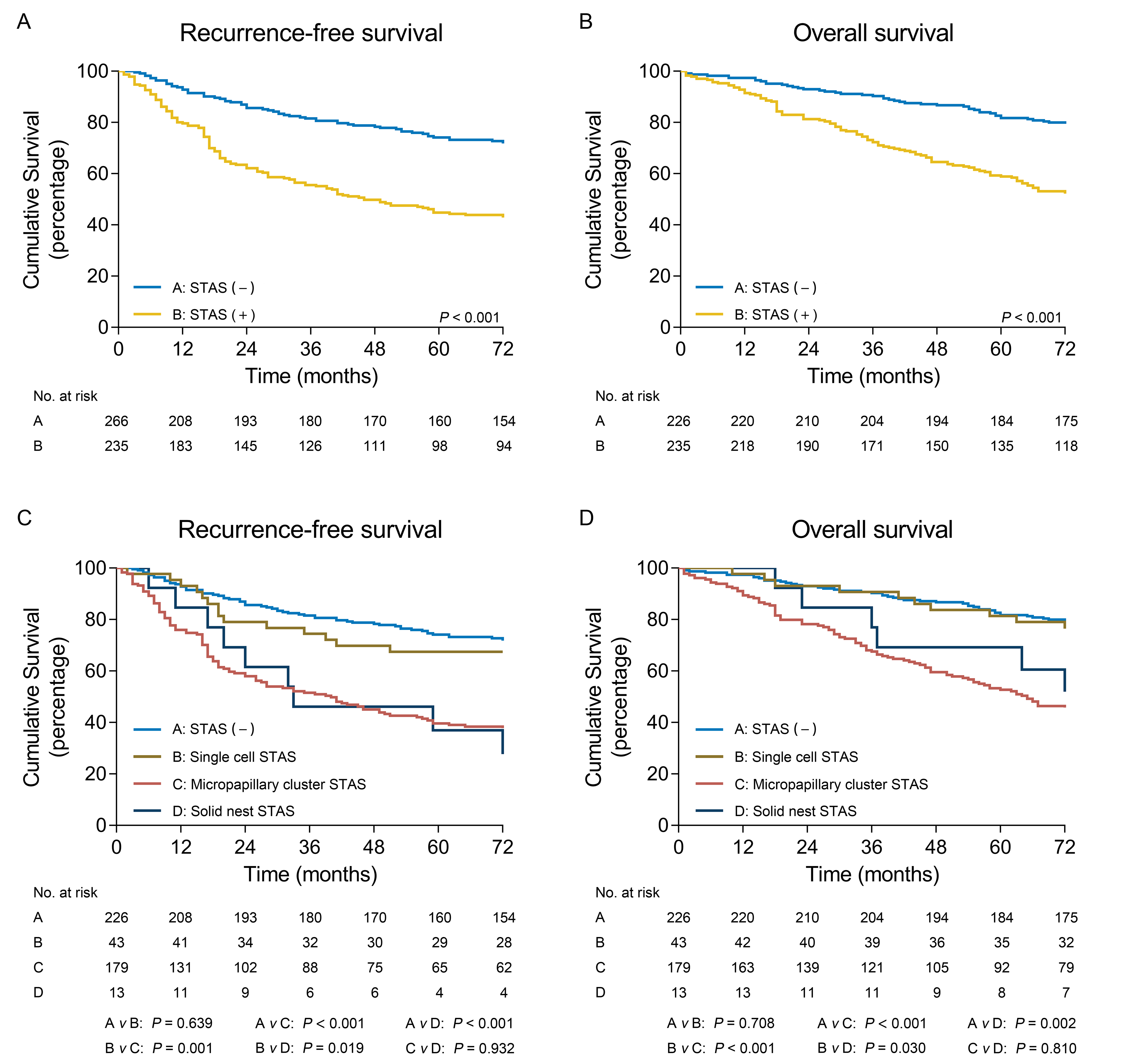

Supplement: Supplementary Figure 1 — Recurrence-free survival (A) and overall survival (B) in patients with adenocarcinoma stratified by STAS. Recurrence-free survival (C) and overall survival (D) in patients with adenocarcinoma stratified by STAS subtypes. STAS, spread through air spaces. [file Image_1.tif]

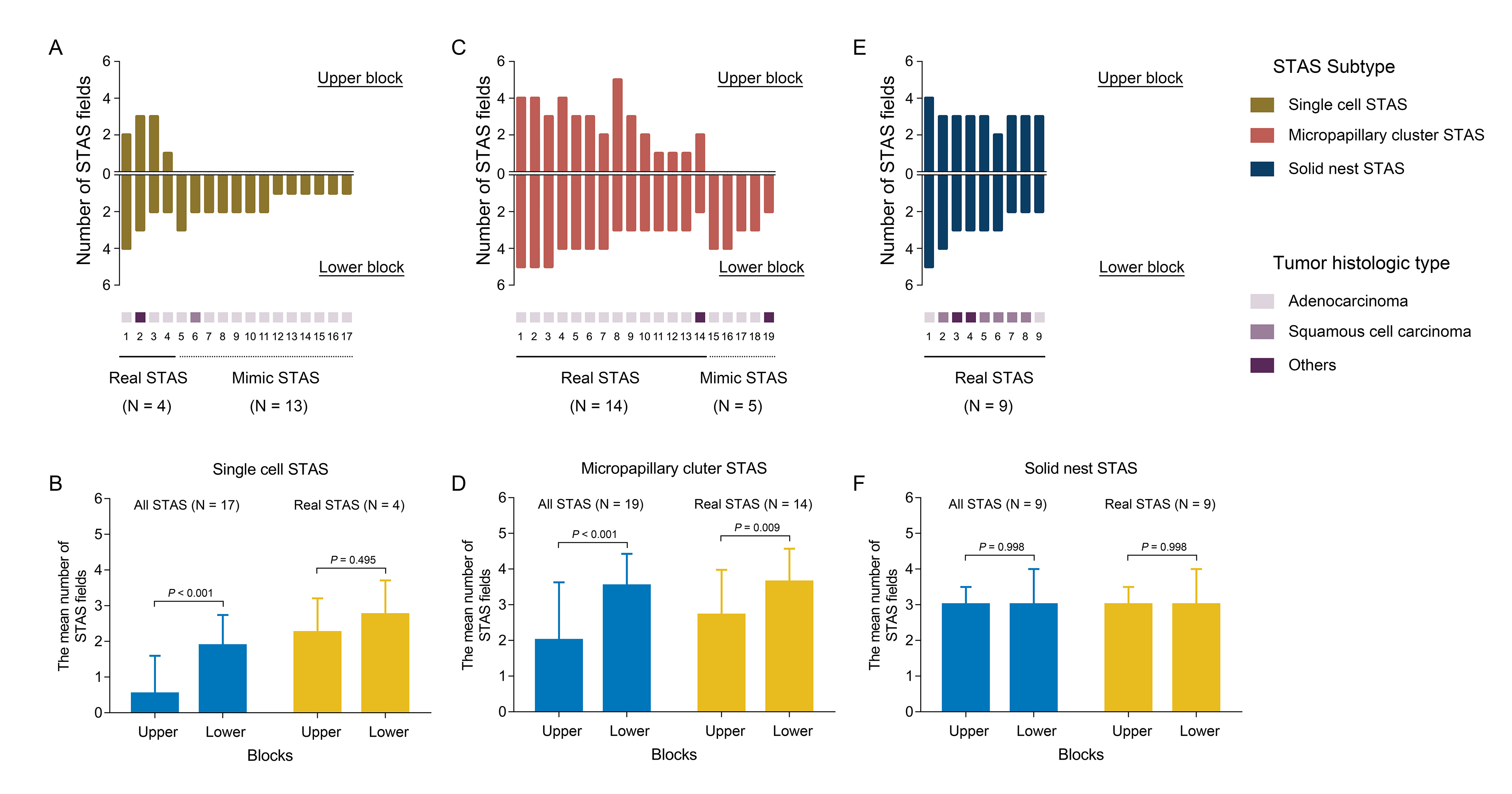

Supplement: Supplementary Figure 2 — The distribution and quantity of STAS subtypes in each tissue block (A, single cell; C, micropapillary cluster; E, solid nest). The quantitative comparison of all STAS subtypes and real STAS subtypes between upper blocks and lower blocks (B, single cell; D, micropapillary cluster; F, solid nest). STAS, spread through air spaces. [file Image_2.tif]
