# Supplementary material for: Morphological Subtypes of Tumor Spread Through Air Spaces in Non-Small Cell Lung Cancer: Prognostic Heterogeneity and Its Underlying Mechanism
Source: Front Oncol. 2021 Mar 4;11:608353. doi: 10.3389/fonc.2021.608353 (PMC7970243; doi:10.3389/fonc.2021.608353)
Supplement: Supplementary file 3 [file Table_1.doc]

Supplementary Table 1. Characteristics of patients with adenocarcinoma stratified by tumor spread through air spaces

| Variables | All patients |  | STAS (-) |  | STAS (+) | *P* value |
| --- | --- | --- | --- | --- | --- | --- |
|  | N = 461 |  | N = 226 |  | N = 235 |  |
| Age |  |  |  |  |  |  |
| Median (range) | 59 (33-90) |  | 59 (37-90) |  | 60 (33-82) | 0.785 |
| ≤ 65 | 318 (69) |  | 155 (69) |  | 163 (69) | 0.857 |
| > 65 | 143 (31) |  | 71 (31) |  | 72 (31) |  |
| Gender |  |  |  |  |  | 0.486 |
| Male | 232 (50) |  | 110 (49) |  | 122 (52) |  |
| Female | 228 (50) |  | 116 (51) |  | 113 (48) |  |
| Smoking |  |  |  |  |  | 0.573 |
| Non-smoker | 350 (76) |  | 169 (75) |  | 181 (77) |  |
| Current or ex-smoker | 111 (24) |  | 57 (25) |  | 54 (23) |  |
| Carcinoembryonic antigen |  |  |  |  |  | < 0.001 |
| Normal | 401 (87) |  | 210 (93) |  | 191 (81) |  |
| High | 60 (13) |  | 16 (7) |  | 14 (19) |  |
| Tumor location |  |  |  |  |  | 0.058 |
| Upper & Middle | 311 (68) |  | 162 (72) |  | 149 (63) |  |
| Lower | 150 (32) |  | 64 (28) |  | 86 (37) |  |
| Surgical type |  |  |  |  |  | 0.001 |
| Limited resection | 27 (6) |  | 9 (4) |  | 18 (8) |  |
| Lobectomy | 413 (89) |  | 214 (95) |  | 199 (84) |  |
| Others | 21 (5) |  | 3 (1) |  | 18 (8) |  |
| Predominant histologic subtype |  |  |  |  |  | < 0.001 |
| Lepidic | 103 (22) |  | 76 (34) |  | 27(12) |  |
| Acinar | 224 (49) |  | 99 (44) |  | 125 (53) |  |
| Papillary | 85 (18) |  | 38 (17) |  | 47 (20) |  |
| Micropapillary | 12 (3) |  | 1 (1) |  | 11 (5) |  |
| Solid | 37 (8) |  | 12 (5) |  | 25 (11) |  |
| Tumor size |  |  |  |  |  | 0.002 |
| ≤ 3 cm | 327 (71) |  | 177 (78) |  | 150 (64) |  |
| > 3-5 cm | 107 (23) |  | 41 (18) |  | 66 (28) |  |
| ≥ 5 cm | 27 (6) |  | 8 (4) |  | 19 (8) |  |
| Visceral pleural invasion |  |  |  |  |  | 0.701 |
| Absent | 251 (54) |  | 121 (54) |  | 130 (55) |  |
| Present | 210 (46) |  | 105 (46) |  | 105 (45) |  |
| Lymph node metastasis |  |  |  |  |  | < 0.001 |
| Negative | 338 (73) |  | 206 (91) |  | 132 (56) |  |
| N1 positive | 29 (6) |  | 5 (2) |  | 24 (10) |  |
| N2 positive | 94 (21) |  | 15 (7) |  | 79 (34) |  |
| Pathologic TNM stage |  |  |  |  |  | < 0.001 |
| Stage I | 310 (67) |  | 193 (85) |  | 117 (50) |  |
| Stage II | 44 (10) |  | 14 (6) |  | 30 (13) |  |
| Stage III/IV | 107 (23) |  | 19 (9) |  | 88 (37) |  |
| STAS Subtype |  |  |  |  |  | - |
| Single cell | 43 (9) |  | - |  | 43 (18) |  |
| Micropapillary cluster | 179 (39) |  | - |  | 179 (76) |  |
| Solid nest | 13 (3) |  | - |  | 13 (6) |  |
| Postoperative chemotherapy |  |  |  |  |  | 0.06 |
| No | 210 (46) |  | 113 (50) |  | 97 (41) |  |
| Yes | 251 (54) |  | 113 (50) |  | 138 (59) |  |

Note: Values are presented as median (range) or n (%). STAS, spread through air spaces.

Supplementary Table 2. Characteristics of patients with adenocarcinoma stratified by subtypes of tumor spread through air spaces

|  | Single cell STAS |  | Micropapillary cluster STAS |  | Solid nest STAS | *P* value |
| --- | --- | --- | --- | --- | --- | --- |
|  | N = 43 |  | N = 179 |  | N = 13 |  |
| Age |  |  |  |  |  |  |
| Median (range) | 59 (35-78) |  | 61 (33-82) |  | 57 (43-79) | 0.323 |
| ≤ 65 | 34 (79) |  | 118 (66) |  | 11 (85) | 0.115 |
| > 65 | 9 (21) |  | 61 (34) |  | 2 (15) |  |
| Gender |  |  |  |  |  | 0.005 |
| Male | 15 (35) |  | 96 (54) |  | 11 (85) |  |
| Female | 28 (65) |  | 83 (46) |  | 2 (15) |  |
| Smoking |  |  |  |  |  | 0.76 |
| Non-smoker | 34 (79) |  | 138 (77) |  | 9 (69) |  |
| Current or ex-smoker | 9 (21) |  | 41 (23) |  | 4 (31) |  |
| Carcinoembryonic antigen |  |  |  |  |  | 0.112 |
| Normal | 37 (86) |  | 141 (79) |  | 13 (100) |  |
| High | 6 (14) |  | 38 (21) |  | 0 (0) |  |
| Tumor location |  |  |  |  |  | 0.445 |
| Upper & Middle | 29 (67) |  | 110 (62) |  | 10 (77) |  |
| Lower | 14 (33) |  | 69 (38) |  | 3 (23) |  |
| Surgical type |  |  |  |  |  | 0.613 |
| Limited resection | 4 (9) |  | 14 (8) |  | 0 (0) |  |
| Lobectomy | 36 (84) |  | 150 (84) |  | 13 (100) |  |
| Others | 3 (7) |  | 15 (8) |  | 0 (0) |  |
| Predominant histologic subtype |  |  |  |  |  | < 0.001 |
| Lepidic | 11 (26) |  | 14 (8) |  | 2 (15) |  |
| Acinar | 17 (40) |  | 104 (58) |  | 4 (31) |  |
| Papillary | 12 (28) |  | 34 (19) |  | 1 (8) |  |
| Micropapillary | 0 (0) |  | 11 (6) |  | 0 (0) |  |
| Solid | 3 (7) |  | 16 (9) |  | 6 (46) |  |
| Tumor size |  |  |  |  |  | 0.233 |
| ≤ 3 cm | 33 (77) |  | 110 (62) |  | 7 (54) |  |
| > 3-5 cm | 6 (14) |  | 55 (31) |  | 5 (38) |  |
| ≥ 5 cm | 4 (9) |  | 14 (7) |  | 1 (8) |  |
| Visceral pleural invasion |  |  |  |  |  | 0.463 |
| Absent | 27 (63) |  | 95 (53) |  | 8 (62) |  |
| Present | 16 (37) |  | 84 (47) |  | 5 (38) |  |
| Lymph node metastasis |  |  |  |  |  | 0.003 |
| Negative | 35 (82) |  | 90 (50) |  | 7 (54) |  |
| N1 positive | 1 (2) |  | 23 (13) |  | 0 (0) |  |
| N2 positive | 7 (16) |  | 66 (37) |  | 6 (46) |  |
| Pathologic TNM stage |  |  |  |  |  | 0.025 |
| Stage I | 30 (70) |  | 81 (45) |  | 6 (46) |  |
| Stage II | 4 (9) |  | 26 (15) |  | 0 (0) |  |
| Stage III/IV | 9 (21) |  | 72 (40) |  | 7 (54) |  |
| Postoperative chemotherapy |  |  |  |  |  | 0.578 |
| No | 20 (47) |  | 73 (41) |  | 4 (31) |  |
| Yes | 23 (53) |  | 106 (59) |  | 9 (61) |  |

Note: Values are presented as median (range) or n (%). STAS, spread through air spaces.

Supplementary Table 3. Cox proportional hazards regression model for recurrence-free survival and overall survival in patients with adenocarcinoma

| Variables | Recurrence-free survival | | | |  | Overall survival | | | |
| --- | --- | --- | --- | --- | --- | --- | --- | --- | --- |
|  | Univariate Analysis |  | Multivariate Analysis | |  | Univariate Analysis |  | Multivariate Analysis | |
|  | *P* value |  | HR (95% CI) | *P* value |  | *P* value |  | HR (95% CI) | *P* value |
| Age |  |  |  |  |  |  |  |  |  |
| > 65 vs. ≤ 65 | 0.294 |  |  |  |  | 0.005 |  | 1.61 (1.18-2.20) | 0.003 |
| Gender |  |  |  |  |  |  |  |  |  |
| Female vs. Male | 0.201 |  |  |  |  | 0.011 |  | 0.72 (0.53-0.98) | 0.037 |
| Smoking |  |  |  |  |  |  |  |  |  |
| Current or ex-smoker vs. Non-smoker | 0.522 |  |  |  |  | 0.199 |  |  |  |
| Carcinoembryonic antigen |  |  |  |  |  |  |  |  |  |
| High vs. Normal | < 0.001 |  | 1.87 (1.31-2.67) | 0.001 |  | < 0.001 |  | 2.10 (1.46-3.01) | < 0.001 |
| Tumor location |  |  |  |  |  |  |  |  |  |
| Lower lobe vs. Upper & middle lobe | 0.213 |  |  |  |  | 0.489 |  |  |  |
| Surgical type |  |  |  |  |  |  |  |  |  |
| Lobectomy & others vs. Limited resection | 0.304 |  |  |  |  | 0.063 |  | 0.43 (0.25-0.77) | 0.004 |
| Tumor histological type | < 0.001 |  |  | 0.116 |  | < 0.001 |  |  | 0.005 |
| Acinar & papillary vs. Lepidic | < 0.001 |  | 1.46 (0.93-2.32) | 0.104 |  | < 0.001 |  | 2.69 (1.46-4.94) | 0.001 |
| Micropapillary & solid vs. Lepidic | < 0.001 |  | 1.84 (1.03-3.30) | 0.039 |  | < 0.001 |  | 2.93 (1.44-5.99) | 0.003 |
| Tumor size | < 0.001 |  |  | 0.078 |  | < 0.001 |  |  | 0.182 |
| 3-5 cm vs. ≤ 3cm | 0.002 |  | 1.15 (0.83-1.59) | 0.416 |  | < 0.001 |  | 1.35 (0.96-1.90) | 0.081 |
| ≥ 5 cm vs. ≤ 3cm | < 0.001 |  | 1.79 (1.07-2.99) | 0.026 |  | 0.001 |  | 1.32 (0.77-2.27) | 0.312 |
| Visceral pleural invasion |  |  |  |  |  |  |  |  |  |
| Present vs. Absent | 0.337 |  |  |  |  | 0.329 |  |  |  |
| Lymph node metastasis |  |  |  |  |  |  |  |  |  |
| Positive vs. Negative | < 0.001 |  | 2.38 (1.71-3.31) | < 0.001 |  | < 0.001 |  | 2.54 (1.78-3.61) | < 0.001 |
| STAS Subtype | < 0.001 |  |  | 0.004 |  | < 0.001 |  |  | 0.012 |
| Single cell STAS vs. Negative | 0.643 |  | 0.82 (0.45-1.49) | 0.517 |  | 0.697 |  | 0.94 (0.48-1.83) | 0.843 |
| Micropapillary cluster STAS vs. Negative | < 0.001 |  | 1.67 (1.18-2.37) | 0.004 |  | < 0.001 |  | 1.73 (1.19-2.51) | 0.004 |
| Solid nest STAS vs. Negative | 0.002 |  | 2.13 (1.02-4.45) | 0.043 |  | 0.002 |  | 2.09 (0.95-4.63) | 0.068 |
| Postoperative chemotherapy |  |  |  |  |  |  |  |  |  |
| Yes vs. No | 0.902 |  |  |  |  | 0.993 |  |  |  |

HR, hazard ratio; CI, confidence interval; STAS, spread through air spaces.

Supplementary Table 4. Baseline characteristics of patients with non-small cell lung cancer in the perspective study

|  | Patients (N = 83) |
| --- | --- |
| Age, years |  |
| Median (range) | 62 (42-81) |
| ≤ 65 | 50 (60) |
| > 65 | 33 (40) |
| Gender |  |
| Male | 54 (65) |
| Female | 29 (35) |
| Smoking |  |
| Non-smoker | 62 (75) |
| Current or ex-smoker | 21 (25) |
| Carcinoembryonic antigen |  |
| Normal | 74 (89) |
| High | 9 (11) |
| Surgical approach |  |
| VATS | 75 (90) |
| Thoracotomy | 8 (10) |
| Operation |  |
| Limited resection | 7 (8) |
| Lobectomy | 76 (92) |
| Tumor location |  |
| Upper & Middle | 50 (60) |
| Lower | 33 (40) |
| Tumor size |  |
| ≤ 3 cm | 65 (78) |
| > 3 cm | 18 (22) |
| Histologic type |  |
| Adenocarcinoma | 68 (82) |
| Squamous cell carcinoma | 9 (11) |
| Others | 6 (7) |
| Lymph node metastasis |  |
| Negative | 70 (84) |
| Positive | 13 (16) |

Values are presented as median (range) or n (%). VATS, video-assisted thoracoscopic surgery.
